# Characterizing and prognosticating chronic lymphocytic leukemia in the elderly: prospective evaluation on 455 patients treated in the United States

**DOI:** 10.1186/s12885-017-3176-x

**Published:** 2017-03-16

**Authors:** Chadi Nabhan, Anthony Mato, Christopher R. Flowers, David L. Grinblatt, Nicole Lamanna, Mark A. Weiss, Matthew S. Davids, Arlene S. Swern, Shriya Bhushan, Kristen Sullivan, E. Dawn Flick, Pavel Kiselev, Jeff P. Sharman

**Affiliations:** 1 Cardinal Health Specialty Solutions, Waukegan, IL 60085 USA; 2 Center for CLL, Abramson Cancer Center, University of Pennsylvania, Philadelphia, PA 19104 USA; 3 Emory University, Atlanta, GA 30322 USA; 4 NorthShore University HealthSystem, Evanston, IL 60201 USA; 5 Leukemia Service, Hematologic Malignancies Section, Division of Hematology/Oncology, New York-Presbyterian Hospital/Columbia University Medical Center, New York, NY 10032 USA; 6 Thomas Jefferson University, Philadelphia, PA 19107 USA; 7 Dana-Farber Cancer Institute, Boston, MA 02215 USA; 8 Celgene Corporation, Summit, NJ 07901 USA; 9 Celgene Corporation, Overland Park, KS 66210 USA; 10 Celgene Corporation, San Francisco, CA USA; 11 Willamette Valley Cancer Institute and Research Center, Springfield, OR USA

**Keywords:** Chronic lymphocytic leukemia, Connect® CLL registry, Elderly, Prognostic, Chemoimmunotherapy

## Abstract

**Background:**

Median age at diagnosis of patients with chronic lymphocytic leukemia (CLL) is > 70 years. However, the majority of clinical trials do not reflect the demographics of CLL patients treated in the community. We examined treatment patterns, outcomes, and disease-related mortality in patients ≥ 75 years with CLL (E-CLL) in a real-world setting.

**Methods:**

The Connect® CLL registry is a multicenter, prospective observational cohort study, which enrolled 1494 adult patients between 2010–2014, at 199 US sites. Patients with CLL were enrolled within 2 months of initiating first line of therapy (LOT1) or a subsequent LOT (LOT ≥ 2). Kaplan–Meier methods were used to evaluate overall survival. CLL- and infection-related mortality were assessed using cumulative incidence functions (CIF) and cause-specific hazards. Logistic regression was used to develop a classification model.

**Results:**

A total of 455 E-CLL patients were enrolled; 259 were enrolled in LOT1 and 196 in LOT ≥ 2. E-CLL patients were more likely to receive rituximab monotherapy (19.3 vs. 8.6%; *p* < 0.0001) and chemotherapy-alone regimens (*p* < 0.0001) than younger patients. Overall and complete responses were lower in E-CLL patients than younger patients when given similar regimens. With a median follow-up of 3 years, CLL-related deaths were higher in E-CLL patients than younger patients in LOT1 (12.6 vs. 5.1% *p* = 0.0005) and LOT ≥ 2 (31.3 vs. 21.5%; *p* = 0.0277). Infection-related deaths were also higher in E-CLL patients than younger patients in LOT1 (7.4 vs. 2.7%; *p* = 0.0033) and in LOT ≥ 2 (16.2 vs. 11.2%; *p* = 0.0786). A prognostic score for E-CLL patients was developed: time from diagnosis to treatment < 3 months, enrollment therapy other than bendamustine/rituximab, and anemia, identified patients at higher risk of inferior survival. Furthermore, higher-risk patients experienced an increased risk of CLL- or infection-related death (30.6 vs 10.3%; *p* = 0.0006).

**Conclusion:**

CLL- and infection-related mortality are higher in CLL patients aged ≥ 75 years than younger patients, underscoring the urgent need for alternative treatment strategies for these understudied patients.

**Trial Registration:**

The Connect CLL registry was registered at clinicaltrials.gov: NCT01081015 on March 4, 2010.

**Electronic supplementary material:**

The online version of this article (doi:10.1186/s12885-017-3176-x) contains supplementary material, which is available to authorized users.

## Background

Chronic lymphocytic leukemia (CLL) accounts for 15 000 diagnosed cases in the USA annually [1]. While incremental improvements in treating CLL have been observed in the past decade [2], the majority of clinical trials leading to these treatment approaches have largely enrolled younger, fitter patients who do not accurately reflect the demographics of CLL patients seen in the community [3–6]. One exception was the CLL-11 study that compared chlorambucil alone to chlorambucil combined with rituximab or obinutuzumab in patients with co-morbidities defined as either a glomerular-filtration rate < 70 mL/min or a cumulative-illness-rating scale ≥ 6 [7]. Other studies have allowed enrollment of elderly patients and performed unplanned subset analyses in an attempt to refine treatments and outcomes in the elderly, but data were inconclusive [8–10]. Moreover, a population-based analysis of 28 590 US patients diagnosed with CLL (1992–2009) showed that the improvement in overall survival (OS) noted in younger patients was less pronounced in the elderly [11]. Furthermore, Brenner et al. [12] showed that improved survival for CLL has not been observed in older patients.

Whether these differences are related to disparities in therapeutic choice, access to care, non-CLL-related deaths in elderly patients, or variations in CLL biology and prognostic indicators is unknown. As the median age of CLL patients at diagnosis approaches 72 years, understanding the biology and outcomes for elderly patients is critical and underscored by the reported inferior survival of these patients.

To examine treatment patterns and disease-related outcomes in elderly CLL patients (defined as ≥ 75 years), we used the Connect® CLL database that enrolled 1494 CLL patients requiring therapy between 2010 and 2014 [13]. These patients were almost entirely enrolled prior to the introduction of novel B-cell receptor (BCR)-targeted therapies. We aimed to establish a benchmark for outcomes in elderly CLL patients treated before the availability of BCR-targeted therapies to help in properly positioning newer agents in the elderly CLL treatment paradigm. Our objective was to compare patient and disease characteristics, prognostic indicators, complications, and disease-related mortality. Further, we aimed to develop a prognostic score that predicts elderly CLL patients at highest risk of CLL- or infection-related deaths. To our knowledge, this represents the largest comprehensive, prospective evaluation of this patient population published to date.

## Methods

### Study design and participants

The Connect CLL registry (NCT01081015), a multicenter, prospective, observational cohort study enrolled 1494 CLL patients treated at 199 US community- and academic-based sites from March 2010 to January 2014 [13]. The study protocol was approved by a central institutional review board (IRB) (Quorum Review IRB, Seattle, WA, USA) or each site’s IRB (Additional file 1). Eligible patients were ≥ 18 years and had CLL as defined by the International Workshop on Chronic Lymphocytic Leukemia (IWCLL) guidelines [14]. Eligible patients were those initiating a first or higher line of therapy (LOT) within 2 months prior to study enrollment. Personnel were educated to enroll patients consecutively as they entered a LOT and to invite every eligible patient to participate in the registry. For this analysis, patients were divided into two groups based on LOT: first line of therapy (LOT1) and second line of therapy or greater (LOT ≥ 2). Each patient was followed up for up to 60 months or until early discontinuation (i.e. due to death, withdrawal of consent, loss to follow-up, or study termination). Follow-up data were collected approximately every 3 months during study participation. Reasons for treatment initiation and responses were assessed by the treating physician.

### Statistical analysis

Date of enrollment was considered baseline for this study. Only laboratory samples collected < 7 days before the start of enrollment therapy were used for baseline laboratory testing. Disease and patients’ characteristics, practice patterns, clinical outcomes, and disease-related mortality were assessed. Continuous variables were reported using measures of dispersion and central tendency (means, medians, ranges, and standard deviation [SD]); categorical variables were reported as numbers and percentages (proportionality, 95% confidence intervals [CI]) of the total study population. Medical history at enrollment and pre-existing condition data were used to generate a Charlson Comorbidity Index (CCI) [15, 16]. Results were summarized by LOT at enrollment (LOT1 or LOT ≥ 2) and by age group (< 75 years and ≥ 75 years). The Chi-square test for the comparison of rates was used to assess differences between patient subgroups. Statistical significance was assessed at *p* = 0.05 (two-sided). The Breslow-Day test was used to assess the homogeneity of the odds ratios.

The Kaplan–Meier method was used to estimate survival, calculated from the date on which therapy was initiated [17]. *p* value was derived from log-rank tests for comparison of survival distributions. CLL-related deaths due to disease progression were distinguished from deaths due to other causes and recorded by the treating physician. CLL- or infection-related survival was assessed using cumulative incidence functions (CIFs); *p* values from Gray’s test for equality of CIFs were reported. Cause-specific hazards analysis identified predictors of survival in univariate and multivariable settings. Predictors demonstrating an association with time to event (*p* < 0.1) were included in multivariable analyses to identify significant independent predictors. Cause-specific hazard ratios (HR) and 95% CI were calculated.

Predictive modeling using logistic regression and a *k*-fold cross-validation method with *k* = 5 was used to develop a prognostic score for elderly CLL patients [18]. Results were confirmed by assessment of the interaction between the above covariates and the elderly CLL group in the analysis of all eligible patients. Statistical analyses were performed using SAS® (version 9.2) statistical software (SAS Institute, Cary, NC, USA).

## Results

### Patient characteristics

Table 1 shows that of 1494 patients enrolled in the registry, 455 patients were ≥ 75 years; 259 patients ≥ 75 years were enrolled in LOT1 and 196 in LOT ≥ 2. Patient demographics and disease characteristics were largely similar between patients enrolled in LOT1 and LOT ≥ 2, with the exception of duration of CLL from diagnosis to enrollment (1.8 vs 7.2 years at LOT ≥ 2). Differences were also observed between patients aged < 75 and ≥ 75 years for Rai staging, constitutional symptoms, and ECOG score at LOT1, and for sex, time from diagnosis to first LOT, race, geographical region, and ECOG score at LOT ≥ 2 (Table 1).

**Table 1:** Demographics and characteristics of patients at enrollment to therapy

	LOT1 (*n* = 889)			LOT ≥ 2 (*n* = 605)		
Characteristics	< 75 years	≥ 75 years	*p* value^a,b^	< 75 years	≥ 75 years	*p* value^a,b^
	(*n* = 630)	(*n* = 259)		(*n* = 409)	(*n* = 196)	
Age, years						
Mean	62.4	80.4		63.9	80.8	
SD	8.26	4.33		7.67	4.37	
Median	63.0	80.0		65.0	80.0	
Range	22–74	75–99		34–74	75–96	
Sex, n (%)						
Male	411 (65.2)	155 (59.8)	**0.1288**	281 (68.7)	106 (54.1)	**0.0005**
Female	219 (34.8)	104 (40.2)		128 (31.3)	90 (45.9)	
Duration of CLL from diagnosis to registry enrollment, years						
Median	1.4	1.8	**0.2912**	7.0	7.2	**0.7074**
Range	0–29	0–32		0–32	0–30	
Time from diagnosis to first LOT, years						
Median	1.4	1.8	**0.2593**	1.4	2.3	**0.0139**
Range	0–29	0–32		0–32	0–20	
Race, n (%)^c,d^						
White	561 (92.3)	237 (92.9)	**0.7211**	352 (90.0)	183 (96.3)	**0.0076**
Black	40 (6.6)	16 (6.3)		37 (9.5)	5 (2.6)	
American Indian/Alaskan native	0	0		1 (0.3)	0	
Asian	3 (0.5)	0		1 (0.3)	0	
Other	4 (0.7)	2 (0.8)		0	2 (1.1)	
Geographic region, n (%)^c,d^						
Northeast	75 (12.0)	37 (14.3)	**0.2029**	58 (14.3)	37 (19.0)	**0.016**
Midwest	207 (33.2)	70 (27.1)		137 (33.7)	45 (23.1)	
South	249 (40.0)	103 (39.9)		162 (39.8)	77 (39.5)	
West	92 (14.8)	48 (18.6)		50 (12.3)	36 (18.5)	
Institution type, n (%)						
Academic	74 (11.7)	12 (4.6)		57 (13.9)	12 (6.1)	
Community	545 (86.5)	242 (93.4)		343 (83.9)	181 (92.3)	
Government	11 (1.7)	5 (1.9)		9 (2.2)	3 (1.5)	
Insurance, n (%)^e^						
Medicare	283 (44.9)	229 (88.4)		227 (55.5)	175 (89.3)	
Medicaid	28 (4.4)	14 (5.4)		16 (3.9)	7 (3.6)	
Supplemental coverage	86 (13.7)	92 (35.5)		81 (19.8)	67 (34.2)	
Private coverage	357 (56.7)	46 (17.8)		189 (46.2)	35 (17.9)	
HMO	88 (14.0)	16 (6.2)		56 (13.7)	13 (6.6)	
PPO	206 (32.7)	26 (10.0)		103 (25.2)	14 (7.1)	
Other	64 (10.2)	4 (1.5)		33 (8.1)	8 (4.1)	
Military	10 (1.6)	5 (1.9)		5 (1.2)	6 (3.1)	
Self-pay	13 (2.1)	0		6 (1.5)	0	
Other Insurance	10 (1.6)	3 (1.2)		8 (2.0)	3 (1.5)	
Not specified	15 (2.4)	5 (1.9)		19 (4.6)	2 (1.0)	
ECOG score and status, n (%)^c,d^						
0 - Fully active	276 (57.4)	70 (33.7)	**<0.001**	138 (46.8)	42 (30.7)	**0.0105**
1 - Restricted in strenuous activity only	180 (37.4)	116 (55.8)		138 (46.8)	79 (57.7)	
2 - Ambulatory, but unable to work	22 (4.6)	19 (9.1)		17 (5.8)	14 (10.2)	
3 - Capable of only limited self-care	2 (0.4)	3 (1.4)		2 (0.7)	2 (1.5)	
4 - Completely disabled	1 (0.2)	0		0	0	
Rai staging system score, n (%)^c,d^						
Stage 0	112 (23.7)	60 (28.4)	**0.0219**	63 (25.2)	46 (32.6)	**0.1728**
Stage I	143 (30.2)	48 (22.7)		58 (23.2)	39 (27.7)	
Stage II	83 (17.5)	25 (11.8)		43 (17.2)	16 (11.3)	
Stage III	71 (15.0)	36 (17.1)		44 (17.6)	24 (17.0)	
Stage IV	64 (13.5)	42 (19.9)		42 (16.8)	16 (11.3)	
Constitutional symptoms, n (%)	397 (63.0)	183 (71.8)	**0.0192**	264 (65.0)	126 (64.3)	**0.977**
Fatigue^f^	328 (82.6)	152 (83.1)		212 (80.3)	114 (90.5)	
Fever	44 (11.1)	16 (8.7)		22 (8.3)	5 (4.0)	
Night sweats	164 (41.3)	62 (33.9)		85 (32.2)	27 (21.4)	
Other	69 (17.4)	44 (24.0)		54 (20.5)	25 (19.8)	
Weight loss	97 (24.4)	60 (32.8)		71 (26.9)	37 (29.4)	
Metaphase cytogenetic analysis, n (%)^e^						
Yes	254 (40.3)	93 (35.9)		148 (36.2)	46 (23.5)	
Abnormalities found^f^	110 (43.3)	48 (51.6)		81 (54.7)	23 (50.0)	
del(11q)	24 (9.4)	12 (12.9)		18 (12.2)	5 (10.9)	
del(13q)	36 (14.2)	11 (11.8)		23 (15.5)	7 (15.2)	
Trisomy 12	41 (16.1)	16 (17.2)		30 (20.3)	8 (17.4)	
del(17p)	13 (5.1)	6 (6.5)		12 (8.1)	3 (6.5)	
Other	35 (13.8)	18 (19.4)		36 (24.3)	10 (21.7)	
FISH analysis, n (%)^e^						
Yes	377 (59.8)	136 (52.5)		157 (38.4)	67 (34.2)	
Abnormalities found^f^	281 (74.5)	99 (72.8)		116 (73.9)	44 (65.7)	
del(11q)	64 (17.0)	26 (19.1)		31 (19.7)	14 (20.9)	
del(13q)	179 (47.5)	59 (43.4)		69 (43.9)	30 (44.8)	
Trisomy 12	74 (19.6)	31 (22.8)		38 (24.2)	10 (14.9)	
del(17p)	33 (8.8)	18 (13.2)		28 (17.8)	9 (13.4)	
Other	24 (6.4)	14 (10.3)		11 (7.0)	6 (9.0)	
ALC (×10^9^/L)	263	106		179	79	
Mean (×10^9^/L)	68.5	58.4		52.7	54.2	
SD	75.7	62.9		64.5	51.9	
Median (×10^9^/L)	46.1	34.6		25.6	40.1	
Range (×10^9^/L)	0–564.0	1.3–275.4		0.1–306.0	0.8–271.1	

^*a*^ *p* values (bold text) calculated using a Chi-square test. ^b^ *p* values of interest are shown. ^c^Data are missing. ^d^Rounding of numbers may cause totals to be =, <, or > 100%. ^e^More than one answer permitted. ^f^Percentages calculated based on the number of patients tested

*ALC* absolute lymphocyte count, *CLL* chronic lymphocytic leukemia, *ECOG* Eastern Cooperative Oncology Group, *FISH* fluorescence in-situ hybridization, *HMO* health maintenance organization, *LOT1* first line of therapy, *LOT ≥ 2* second line of therapy or greater, *PPO* preferred provider organization, *SD* standard deviation

### Treatment patterns

Elderly CLL patients were more likely to receive rituximab monotherapy than younger patients, regardless of LOT (19.3 vs. 8.6% in LOT1; 15.3 vs. 12.7% in LOT ≥ 2). This was significant for patients receiving LOT1 (*p* < 0.0001) (Table 2). Patients ≥ 75 years in LOT ≥ 2 were significantly less likely to receive bendamustine/rituximab (BR) than patients < 75 years (21.9 vs. 30.6%; *p* = 0.0267). Only 6.9% of patients ≥ 75 years in LOT1 received fludarabine/cyclophosphamide/rituximab (FCR), versus 33.7% of patients < 75 years (*p* < 0.0001). Interestingly, patients ≥ 75 years were significantly more likely to receive chemotherapy alone without anti-CD20 antibody therapy than patients < 75 years. This was true for LOT1 (20.1 vs. 10.3%; *p* < 0.0001) and LOT ≥ 2 (25.5 vs. 11.0%; *p* < 0.0001).

**Table 2:** Type of therapy by age group (most frequently used regimens)

Regimen	LOT1 (*n* = 889)			LOT ≥ 2 (*n* = 605)		
	< 75 years	≥ 75 years	*p* value^a,b^	< 75 years	≥ 75 years	*p* value^a,b^
	(*n* = 630)	(*n* = 259)		(*n* = 409)	(*n* = 196)	
Rituximab monotherapy, n (%)	54 (8.6)	50 (19.3)	**<0.0001**	52 (12.7)	30 (15.3)	**0.3834**
Combinations with rituximab, n (%)	482 (76.5)	139 (53.7)	**<0.0001**	243 (59.4)	90 (45.92)	**0.0018**
Bendamustine/rituximab	126 (20.0)	61 (23.6)	**0.2377**	125 (30.6)	43 (21.9)	**0.0267**
Bendamustine/dexamethasone/rituximab	5 (0.8)	4 (1.5)		5 (1.2)	0	
Chlorambucil/rituximab	0	4 (1.5)		0	0	
Cyclophosphamide/rituximab	0	0		0	3 (1.5)	
Cyclophosphamide/fludarabine/dexamethasone/rituximab	8 (1.3)	0		0	0	
Cyclophosphamide/lenalidomide/rituximab	0	0		4 (1.0)	0	
Cyclophosphamide/pentostatin/rituximab	21 (3.3)	3 (1.2)		13 (3.2)	0	
Cyclophosphamide/vincristine/prednisone/rituximab	14 (2.2)	9 (3.5)		8 (2.0)	6 (3.1)	
Fludarabine/cyclophosphamide/rituximab	212 (33.7)	18 (6.9)	**<0.0001**	41 (10.0)	11 (5.6)	**0.0700**
Fludarabine/rituximab	33 (5.2)	22 (8.5)		14 (3.4)	11 (5.6)	
Lenalidomide/rituximab	10 (1.6)	0		0	0	
Prednisone/rituximab	0	4 (1.5)		0	3 (1.5)	
Investigational product/rituximab	14 (2.2)	0		0	0	
Chemotherapy alone, n (%)	65 (10.3)	52 (20.1)	**<0.0001**	45 (11.0)	50 (25.5)	**<0.0001**
Bendamustine	23 (3.7)	8 (3.1)		24 (5.9)	21 (10.7)	
Chlorambucil	18 (2.9)	22 (8.5)		6 (1.5)	12 (6.1)	
Chlorambucil/prednisone	0	12 (4.6)		0	3 (1.5)	
Cyclophosphamide	0	4 (1.5)		0	0	
Cyclophosphamide/fludarabine	11 (1.7)	0		0	2 (1.0)	
Cyclophosphamide/vincristine/prednisone	0	0		5 (1.2)	0	
Fludarabine	9 (1.4)	0		5 (1.2)	10 (5.1)	
Other, n (%)	14 (2.2)	16 (6.2)	**0.0030**	46 (11.3)	18 (9.2)	**0.4400**
Alemtuzumab	0	0		14 (3.4)	6 (3.1)	
Lenalidomide	0	4 (1.5)		0	0	
Ofatumumab	0	0		17 (4.2)	8 (4.1)	
Investigational product	6 (1.0)	7 (2.7)		13 (3.2)	4 (2.0)	

^a^ *p* values (bold text) calculated using a Chi-square test. ^b^ *p* value shown for large patient groups only

*LOT1* first line of therapy, *LOT ≥ 2* second line of therapy or greater

Geographic variations in treatment patterns were also observed. In elderly CLL patients in LOT1, the South had the highest utilization of rituximab-based regimens (61.2%) while the West had the lowest (29.2%; *p* < 0.0023). For patients covered by private insurance, younger CLL patients were more likely to receive rituximab-based therapies than elderly CLL patients (80.1 vs. 50.0%; *p* < 0.0001). This was also observed for patients covered by other insurance providers including Medicare, Medicaid, and military health insurance (71.8 vs. 54.5%; *p* < 0.0001). When analyzed using the Breslow-Day test, the results did not differ significantly by health insurance coverage (*p* = 0.0879).

### Response and survival

For all patients enrolled in LOT1, overall response rate (ORR) was 60.2% (38.1% complete response [CR]) while patients enrolled in LOT ≥ 2 had an ORR of 42.6% (17.0% CR). In LOT1, ORRs were significantly lower in patients ≥ 75 years compared with patients < 75 years (ORR: 48.3 vs. 65.1% respectively; *p* < 0.0001 and CR: 25.9 vs. 42.3%, respectively; *p* < 0.0001). Lower ORR and CR were also observed for elderly CLL patients in LOT1 when specific enrollment therapies were analyzed (Additional file 2: Table S1). Similarly, lower ORRs were observed in LOT ≥ 2 (CR: 11.2 vs. 19.8%; *p* = 0.009). As responses were investigator-assessed, we investigated whether patients were evaluated by imaging at enrollment. Patients ≥ 75 years were less likely to be evaluated by imaging than patients < 75 years (65.4 vs. 72.0%; *p* = 0.004). This finding was maintained after adjusting for LOT.

### Outcomes

**Fig. 1:**
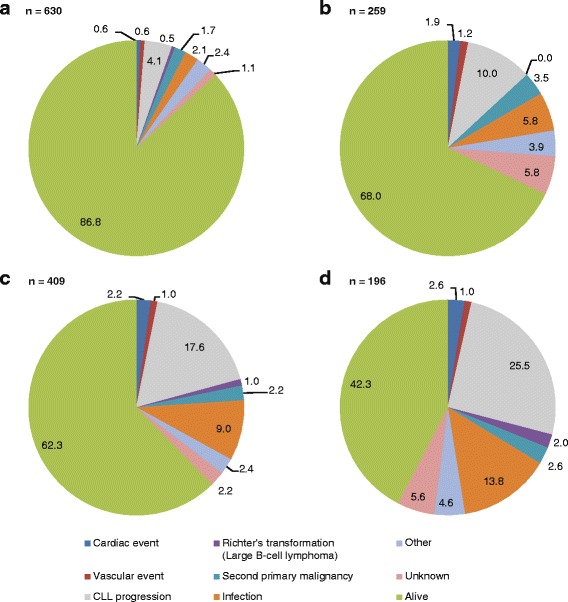
Cause of death among patients enrolled on the registry. Cause of death is shown for **a** patients aged < 75 years in LOT1; **b** patients aged ≥ 75 years in LOT1; **c** patients aged < 75 years in LOT ≥ 2; **d** patients aged ≥ 75 years in LOT ≥ 2. Rounding of values may cause totals to be equal, >, or < 100%. CLL chronic lymphocytic leukemia, LOT1 first line of therapy, LOT ≥ 2 second line of therapy or greater

**Fig. 2:**
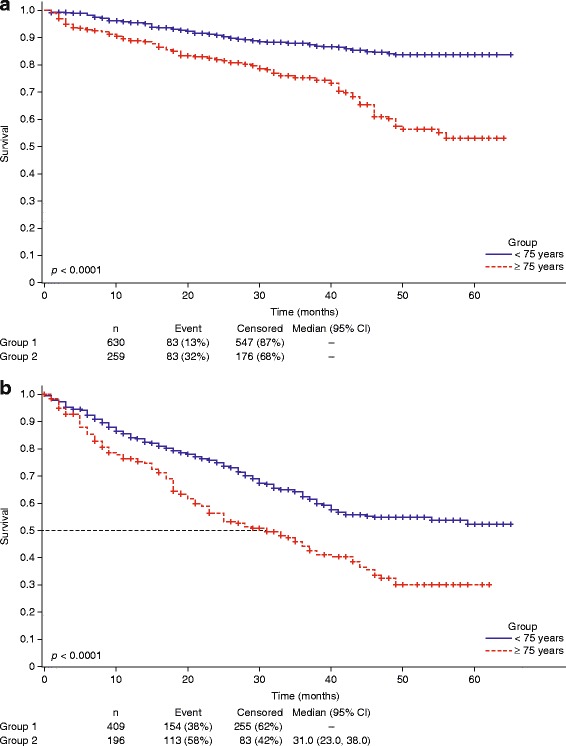
Overall survival in elderly CLL patients vs. younger patients. Kaplan–Meier curves of OS for patients in **a** LOT1 and **b** LOT ≥ 2 stratified by age. Percentages are rounded to the nearest whole number. CI confidence interval, LOT1 first line of therapy, LOT ≥ 2 second line of therapy or greater, OS overall survival

As of August 25, 2015, with a median follow-up of 32.6 months for all 1494 patients, 433 (29%) had died; causes of death are summarized in Fig. 1. As expected, OS was significantly lower in patients ≥ 75 years than patients < 75 years in both LOT1 (log-rank *p* < 0.0001; Fig. 2a) and LOT ≥ 2 (log-rank *p* < 0.0001; Fig. 2b).

**Fig. 3:**
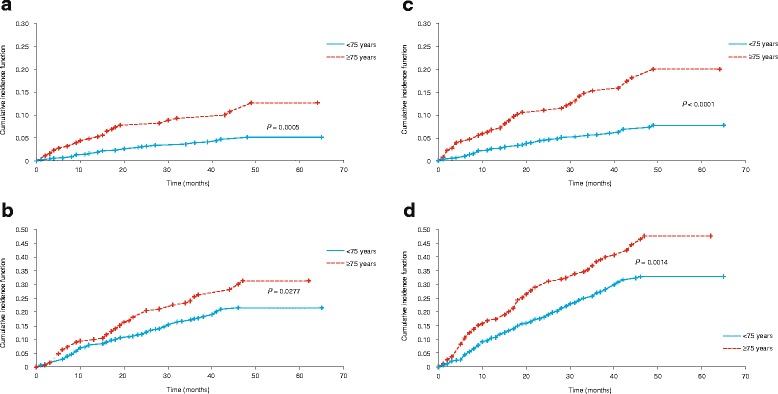
Cumulative incidence of deaths in elderly CLL patients vs. younger patients. CIF of CLL-related deaths stratified by age in **a** LOT1 and **b** LOT ≥ 2, and CLL- or infection-related deaths stratified by age in **c** LOT1 and **d** LOT ≥ 2, demonstrating increased mortality in elderly CLL patients (*red line*). Horizontal dashed line shows median survival in patients ≥ 75 years. CI confidence interval, CIF cumulative incidence functions, CLL chronic lymphocytic leukemia, LOT1 first line of therapy, LOT ≥ 2 second line of therapy or greater

Notably, elderly CLL patients were more likely to die from CLL in LOT1 (12.6 vs. 5.1%, Gray’s test *p* = 0.0005; Fig. 3a) and LOT ≥ 2 (31.3 vs. 21.5%, Gray’s test *p* = 0.0277; Fig. 3b). Time to death from CLL or infection in patients in LOT1 was also significantly shorter in patients ≥ 75 years than patients < 75 years (Gray’s test *p* < 0.0001; Fig. 3c), and in patients in LOT ≥ 2 (Gray’s test *p* = 0.0014; Fig. 3d). Analysis of cause-specific hazards was performed to identify predictors of death from CLL in patients enrolled in LOT1. In univariate analyses, insurance status, anemia, del(17p) abnormality, and age ≥ 75 years (Additional file 3: Table S2) were identified as significant factors. Multivariable analysis retained age ≥ 75 years at enrollment (HR: 3.66, 95% CI 1.92–7.00), and the presence of the del(17p) abnormality (by fluorescence in situ hybridization or cytogenetic testing) (HR: 2.63, 95% CI 1.20–5.78) as independent predictors of a higher risk of death.

### Prognostic model for early death from CLL or infection in elderly CLL patients

We performed prognostic modeling on 181 elderly CLL patients receiving LOT1 who were followed up for ≥ 2 years. Modeling was carried out using the *k*-fold cross-validation method. Due to the limited sample size, a 5-fold cross-validation approach was chosen. The sample of 181 patients was randomly partitioned into five validation subsets of approximately equal size. Five models were generated using this approach. In multivariable analyses, significant predictors of death due to CLL or infection included choice of enrollment therapy, CCI score, time from diagnosis, anemia at enrollment, sex, and race. However, validation of these models did not provide consistent results primarily due to the small size of the validation datasets. Therefore, a decision was made to identify independent predictors of death among the covariates that were part of at least one multivariable model. These covariates were used in the final model.

Three predictors were identified: time from diagnosis to first treatment, enrollment therapy other than BR, and anemia. Based on the relative magnitude of effect, each predictor was weighted and assigned a score [19]. Time from diagnosis to treatment < 3 months and therapy other than BR were assigned a score of 2; anemia at enrollment was assigned a score of 1. Patients were classified into risk groups: lower-risk (score ≤ 4) and higher-risk (score = 5). When stratified by risk, mortality due to CLL or infection was 10.3% in the lower-risk group (*n* = 145) compared with 30.6% in the higher-risk group (*n* = 36) (Chi-square *p* = 0.0002). This prognostic model was validated in a multivariate analysis of all patients with a grouping variable and interaction terms for each of the significant covariates.

### Serious adverse events

Serious adverse events (SAEs) of any grade were more common in patients ≥ 75 years than patients < 75 years in LOT1 (56.0 vs. 39.4%) and LOT ≥ 2 (68.4 vs. 61.9%) (Additional file 4: Table S3). Grade ≥ 3 SAEs were more common in elderly CLL patients in LOT1 (51.4 vs. 34.8%) (Additional file 5: Table S4). The most frequent grade ≥ 3 SAE, pneumonia, was more common in elderly CLL patients in LOT1 (9.7%) than in patients < 75 years (4.0%); however, in LOT ≥ 2 rates of grade ≥ 3 pneumonia were similar in both groups (12.8 vs. 13.7%, respectively). In LOT ≥ 2, febrile neutropenia, thrombocytopenia, and pyrexia were more common in patients < 75 years (Additional file 4: Table S3 and Additional file 5: Table S4).

## Discussion

While inferior OS is expected in elderly CLL patients, our analysis of elderly CLL patients treated in a ‘real-world’ setting showed that these patients are more likely to experience CLL- or infection-related deaths. To our knowledge, this has not been reported previously. We developed a prognostic score specifically for this vulnerable patient population, which classified the elderly CLL cohort into high- and low-risk groups with statistical variation in CLL-related mortality.

As the US population ages, identifying optimal therapeutic strategies for the elderly is a critical unmet medical need as few prospective trials have targeted this patient population. Moreover, elderly patients enrolled in clinical trials might not represent the general elderly population treated in the community. While geriatric assessments should be used to provide an objective and comparable measure of elderly status [20], most studies define elderly patients based solely on an age cut-off. As the median age at diagnosis is 72 years, we selected ≥ 75 years of age as the cut-off for this analysis. While there are limitations to selecting an age cut-off, we postulated that a cut-off above the median age at diagnosis would be clinically meaningful. In addition, published prospective data on outcomes for patients who are ≥ 75 years of age are limited [21, 22].

Elderly CLL patients were more often treated with rituximab monotherapy than their younger counterparts who were more likely to receive chemoimmunotherapy [22]. However, the fact that 20% of elderly CLL patients did not receive an anti-CD20 monoclonal antibody is striking, given that all patients were treated after 2010. Even in the younger cohort, we observed that 10% of patients did not receive any anti-CD20 antibodies. To better understand this variation, we assessed whether patterns of care differed based on health insurance coverage or geographic location of the treating institution. Elderly CLL patients were less likely to receive rituximab-based therapies than younger patients, regardless of insurance provider. However, patients residing in the South were more likely to receive anti-CD20 therapy compared with patients living on the West coast. A comparable observation was reported in a study of follicular lymphoma patients in the West of the USA who were less likely to receive rituximab-based maintenance therapy [23]. This may reflect differences in the treating institution and/or setting. Rituximab use has increased in hospitals while declining in clinics, which could account for the imbalance in treatment between geographic locations [24]. These data suggest that real-world findings differ from clinical trial observations.

Regardless of LOT, responses appeared lower in elderly CLL patients. Although responses were assessed by treating physicians and were not centrally reviewed, CR in the younger patients at LOT1 (42.3%) was comparable to the response (44%) reported for treatment-naïve patients in the CLL-8 trial of rituximab plus fludarabine/cyclophosphamide [6]. Only 25.9% of elderly CLL patients achieved a CR in LOT1. Given the association between survival and the depth of remission [25], this finding is critical and might contribute to the inferior outcomes noted in our elderly cohort.

Despite the typically indolent nature of CLL, we observed critical outcome differences at a median follow-up of 32.6 months. OS was inferior in elderly CLL patients in any LOT group. Given the predictably inferior OS in the elderly due to competing co-morbidities and deaths from other causes, we compared CLL-related deaths between both groups in LOT1 and LOT ≥ 2. Only 5% of patients < 75 years in LOT1 experienced CLL-specific deaths while 13% of elderly CLL patients died from CLL alone. This difference was statistically significant (*p* = 0.0005). A similar observation was noted in LOT ≥ 2 (31% for ≥ 75 years vs. 22% for < 75 years; *p* = 0.0277). Since infections are a major cause of CLL-related deaths, we evaluated the differences in deaths due to CLL or infection in both LOT groups. The difference remained significant (*p* < 0.0001 in LOT1; *p* = 0.0014 in LOT ≥ 2).

We subsequently studied prognostic indicators for CLL- or infection-related deaths in elderly CLL patients. We identified three factors that were significant in a multivariable analysis: time from diagnosis to therapy initiation of < 3 months, enrollment therapy other than BR, and anemia. While a time from diagnosis to therapy of < 3 months may suggest patients had more aggressive disease, this is not necessarily related to disease staging. Indeed, the majority of patients in each LOT and age group had Rai stage 0–2. Rai stage did also not differ significantly between younger and older patients. The prognostic score was used to classify elderly CLL patients according to high- or low-risk of CLL-related death (30.6 vs. 10.3%, respectively; *p* = 0.0002). Contrary to the prognostic models published by Pflug et al. [26], and The International Prognostic Index for patients with CLL (CLL-IPI) working group [27] in which all patients were included regardless of age, our score was specifically designed for elderly CLL patients. Notably, Pflug et al. [26] and the CLL-IPI working group [27] identified older age as an independent factor negatively impacting survival. Our model is also specific to patients receiving therapy as patients under observation were not enrolled to the registry.

Several limitations inherent in any registry-based observational study were encountered during our study. These include the non-random allocation of patients to specific interventions, the assessment of outcomes by non-blinded individuals, and the greater potential for missing data [28]. In the Connect CLL registry, responses were not centrally assessed and indications to treat were based on the treating physician’s judgment. Comprehensive molecular and cytogenetic evaluation was missing for some patients. Our analysis also has limitations that are specific to the Connect CLL registry. Only patients requiring therapy were enrolled in the registry. Patients who died without starting therapy were excluded. The registry predates the introduction of BCR-targeted therapies; therefore, the patients in this registry were not treated with these novel agents. As with any registry, patients were treated with a number of different therapies. The small size of the cohort and the inclusion of only 181 patients in the prognostic model may also be limiting factors. However, despite the small sample size we believe that these results are meaningful as they relate specifically to elderly patients. Importantly, these data also represent the largest US population of CLL patients treated outside of interventional clinical trials in the chemoimmunotherapy era.

Our finding of increased mortality related to elderly CLL patients highlights the urgent need for therapies tailored to this population and underscores the need to refine CLL treatment for the elderly as current therapies and strategies appear suboptimal. This might reflect a limited enrollment of elderly patients into clinical trials and highlight a flaw in the assumption that effective regimens in younger patients will be effective in elderly patients. As new BCR-targeted agents are increasingly used, their role in elderly CLL patient treatment will require critical analysis to balance efficacy with toxicity. Our data on CLL- and infection-related mortality using traditional therapies are a benchmark against which novel therapies can be measured. Finally, the proposed prognostic score, while requiring validation in patients treated with BCR-targeted therapies, could be used to stratify elderly CLL patients on their enrollment into future clinical trials.

## Conclusion

These data represent the real-world experiences of a large population of CLL patients treated across the USA. Within the limitations of an observational registry we have shown that elderly CLL patients have inferior outcomes with a cumulative increased risk of death from CLL regardless of LOT. Recent improvements in survival for younger patients with CLL have still to be achieved in elderly CLL patients. While elderly people have increased mortality versus younger people regardless of CLL status, it will be important to identify new therapies that can improve the outcomes for elderly CLL patients, similar to the advances seen in younger CLL patients. This unique prognostic model for patients ≥ 75 years could identify those patients who would benefit from early treatment or treatment with novel therapies.

## Additional files

Additional file 1: List of site-specific IRBs. (DOCX 22 kb)
Additional file 2: Table S1. CR and ORR of patients < 75 years versus patients ≥ 75 years enrolled in LOT1 by specific therapy. (DOCX 17 kb)
Additional file 3: Table S2. Cause-specific hazards analysis of prognostic indicators for OS. (DOCX 18 kb)
Additional file 4: Table S3. Incidence of serious adverse events of any grade in enrolled patients by therapy and age group. (DOCX 18 kb)
Additional file 5: Table S4. Incidence of serious adverse events of grade ≥ 3 in enrolled patients by therapy and age group. (DOCX 18 kb)

## Abbreviations

BCR: B cell receptor
BR: Bendamustine and rituximab
CCI: Charlson comorbidity index
CI: Confidence interval
CIF: Cumulative incidence function
CLL: Chronic lymphocytic leukemia
CR: Complete response
E-CLL: Elderly CLL
FCR: Fludarabine, cyclophosphamide, and rituximab
HR: Hazard ratios
IRB: Institutional review board
IWCLL: International Workshop on Chronic Lymphocytic Leukemia
LOT: Line of therapy
ORR: Overall response rate
OS: Overall survival
SAEs: Serious adverse events
SD: Standard deviation
