# Supplementary material for: Characterizing and prognosticating chronic lymphocytic leukemia in the elderly: prospective evaluation on 455 patients treated in the United States
Source: BMC Cancer. 2017 Mar 16;17:198. doi: 10.1186/s12885-017-3176-x (PMC5356242; doi:10.1186/s12885-017-3176-x)
Supplement: Additional file 1: — List of site-specific IRBs. (DOCX 22 kb) [file 12885_2017_3176_MOESM1_ESM.docx]

| HSHS St. Vincent Hospital Regional Cancer Center | 835 S Van Buren Street | Green Bay | Wisconsin | 54301 |
| --- | --- | --- | --- | --- |
| UW Cancer Center at Pro Health Care | N16W24131 Riverwood Drive | Waukesha | Wisconsin | 53188 |
| PeaceHealth St. Joseph Medical Center | 2901 Squalicum Parkway | Bellingham | Washington | 98225 |
| Cancer Care Northwest - Holland | 605 E. Holland | Spokane | Washington | 99218 |
| Cancer Care Northwest - Mission | 12615 E. Mission | Spokane | Washington | 99202 |
| Cancer Care Northwest - Sherman | 601 S. Sherman | Spokane | Washington | 99202 |
| Puget Sound Cancer Center- Edmonds | 21605 76th Avenue West | Edmonds | Washington | 98026 |
| Puget Sound Cancer Center- Seattle | 1560 North 115th Street | Seattle | Washington | 98133 |
| Yakima Valley Memorial Hospital/North Star Lodge | 808 North 39th Avenue | Yakima | Washington | 98902 |
| Fairfax Northern Virginia Hematology-Oncology PC- Arlington | 1635 North George Mason Drive | Arlington | Virginia | 22205 |
| Fairfax Northern Virginia Hematology-Oncology PC | 8503 Arlington Blvd. | Fairfax | Virginia | 22031 |
| Fairfax Northern Virginia Hematology-Oncology PC- Gainesville | 7901 Lake Manassas Drive | Gainesville | Virginia | 20155 |
| Fairfax Northern Virginia Hematology-Oncology PC- Leesburg | 44035 Riverside Pkwy Suite 300 | Leesburg | Virginia | 20176 |
| Oncology and Hematology Assoc. of SW VA, Inc.- Raonoke | 2013 S. Jefferson St., 2nd Floor | Roanoke | Virginia | 24014 |
| Oncology and Hematology Assoc. of SW VA, Inc.- Christianburg | 2955 Market Street | Christiansburg | Virginia | 24073 |
| Oncology and Hematology Assoc. of SW VA, Inc.- Salem | 1900 Electric Road, First Floor | Salem | Virginia | 24153 |
| Oncology and Hematology Assoc. of SW VA, Inc.- Wytheville | 2013 S. Jefferson Street | Roanake | Virginia | 24014 |
| Shenandoah Oncology | 400 Campus Boulevard, Suite 100 | Winchester | Virginia | 22601 |
| Baylor Scott & White Health | 302 University Blvd | Round Rock | Texas | 78665 |
| Joe Arrington Cancer Research Treatment Center | 4101 22nd Place | Lubbock | Texas | 79410 |
| Brooke Army Medical Center (BAMC) | 3551 Roger Brooke Drive | Fort Sam Houston | Texas | 78234 |
| sSt. Joseph Cancer Center | 2215 East Villa Maria | Bryan | Texas | 77802 |
| University of Texas Medical Branch at Galveston | 301 University Blvd. | Galveston | Texas | 77555 |
| Texas Oncology, P.A. - Midland | 400 North Garfield | Midland | Texas | 79701 |
| Cancer Care Network of South Texas - SAT&BC | 2130 NE Loop 410 | San Antonio | Texas | 78217 |
| El Paso Cancer Treatment Center- West | 1901 Grandview Avenue | El Paso | Texas | 79902 |
| Cancer Care Centers of South Texas | 694 Hill County Drive | Kerrville | Texas | 78028 |
| Cancer Care Network of South Texas - SAT&BC-M oak | 540 Madison Oak, Suite 200 | San Antonio | Texas | 78258 |
| Southwest Fort Worth Cancer Center | 6500 Harris Parkway | Fort Worth | Texas | 76132 |
| Texas Oncology, P.A. - Abilene | 1957 Antilley Rd. | Abilene | Texas | 79606 |
| Texas Oncology- Arlington South | 515 W. Mayfield Rd. #101 | Arlington | Texas | 76014 |
| Texas Oncology, P.A. - McAllen | 1901 South 2nd Street | McAllen | Texas | 78503-1298 |
| Texas Oncology, P.A. - Mesquite | 4700 N. Galloway Avenue | Mesquite | Texas | 75150 |
| Texas Oncology, P.A. - Paris | 3550 Northeast Loop 286 | Paris | Texas | 75460 |
| Texas Oncology, P.A. - Sherman | 2800 Highway 75 North | Sherman | Texas | 75090 |
| Texas Oncology, P.A. - Tyler | 910 E. Houston Street | Tyler | Texas | 75702 |
| Texas Oncology, P.A. - Weslaco | 1330 East 6th Street | Weslaco | Texas | 78596 |
| Texas Oncology, P.A. - Waco | 1700 W. Hwy. 6 | Waco | Texas | 76712 |
| Texas Oncology, P.A. - Amarillo | 1000 S. Coulter, Suite 100 | Amarillo | Texas | 79106 |
| Texas Oncology, P.A. - Dallas Presbyterian | 8220 Walnut Hill Lane | Dallas | Texas | 75231 |
| Texas Oncology, P.A. - Fort Worth | 1001 12th Ave | Fort Worth | Texas | 76104 |
| Texas Oncology, P.A. - Wichita Falls | 5400 Kell West Blvd. | Wichita Falls | Texas | 76310 |
| SCRI - Tennessee Oncology | 3322 West End Avenue | Nashville | Tennessee | 37203 |
| Sanford Research / USD-Sioux Falls | 1018 West 18th Street | Sioux Falls | South Dakota | 57104 |
| Cancer Centers of the Carolinas - Seneca | 131 Lila Doyle Drive | Seneca | South Carolina | 29672 |
| Cancer Centers of the Carolinas- Eastside | 65 International Drive | Greenville | South Carolina | 29615 |
| MD Anderson Cancer Center | 1515 Holcombe Boulevard, Unit 430 | Houston | Texas | 77030 |
| Bay Regional Medical Center | 1900 Columbus Avenue | Bay City | Michigan | 48708 |
| Spartanburg Regional Healthcare System | 101 East Wood Street | Spartanburg | South Carolina | 29303 |
| Thomas Jefferson University Hospital | 1015 Chestnut St, Suite 320A | Philadelphia | Pennsylvania | 19107 |
| Geisinger Health System | 100 North Academy Avenue | Danville | Pennsylvania | 17822 |
| Drexel University College of Medicine | 245 North 15th Street | Philadelphia | Pennsylvania | 19102 |
| Medical Oncology Associates | 382 Pierce Street | Kingston | Pennsylvania | 18704 |
| Northwest Cancer Specialists PC- Barnes | 9555 SW Barnes Road | Portland | Oregon | 97225 |
| Northwest Cancer Specialists PC- Tualatin | 19260 SW 65th Ave., Suite 435 | Tualatin | Oregon | 97062 |
| Oncology Associates of Oregon PC- Eugene | 520 Country Club Road | Eugene | Oregon | 97401 |
| Oncology Associates of Oregon PC- Springfield | 3377 RiverBend Drive | Springfield | Oregon | 97477 |
| Summa Health System | 525 E Market Street | Akron | Ohio | 44304 |
| Roger Maris Cancer Center | 820 4th Street North | Fargo | North Dakota | 58122-002 |
| Cancer Centers of North Carolina – Cary | 216 Ashville Ave | Cary | North Carolina | 27518 |
| Cancer Centers of North Carolina - Macon | 4101 Macon Pond Rd | Raleigh | North Carolina | 27607 |
| Stratton Veterans Affairs Medical Center - Albany | 113 Holland Ave. | Albany | New York | 12208 |
| Somerset Hematology Oncology Associates | 30 Rehill Avenue, Suite 2200 | Somerville | New Jersey | 08876 |
| Hackensack University Medical Center | 92 Second St | Hackensack | New Jersey | 07601 |
| VA Sierra Nevada Healthcare System | 975 Kirman Avenue | Reno | Nevada | 89502 |
| Mercy Research | 524 North Boonville | Springfield | Missouri | 65806 |
| Mercy Research | 607 South New Ballas Road, Suite 3425 | St. Louis | Missouri | 63141 |
| Health Midwest Ventures Group, Inc d/b/a HCA MidAmerica Division, LLC | 2316 E. Meyer Blvd. | Kansas City | Missouri | 64132 |
| Missouri Cancer Associates | 1705 E Broadway | Columbia | Missouri | 65201 |
| Park Nicollet Institute | 3931 Louisiana Avenue, South | St. Louis Park | Minnesota | 55426 |
| Park Nicollet Institute | 3800 Park Nicollet Blvd | Saint Louis Park | Minnesota | 55416 |
| Duluth Clinic, Ltd. | 400 East Third Street | Duluth | Minnesota | 55805 |
| West Michigan Cancer Center | 200 N. Park Street | Kalamazoo | Michigan | 49007 |
| McLaren Regional Medical Center | 4100 Beecher, Suite B | Flint | Michigan | 48532 |
| Michigan State University | 401 W. Greenlawn Avenue | Lansing | Michigan | 48910 |
| LaPeer Regional Medical Center | 1295 W Berry Drive | Lapeer | Michigan | 48446 |
| Mount Clemens Regional Medical Center | 1080 Harrington Blvd | Mount Clemens | Michigan | 48043 |
| Davenport Mugar Cancer Center | 27 Park St | Hyannis | Massachusetts | 02601 |
| Reliant Medical Group | 1 Eaton Place | Worcester | Massachusetts | 01608 |
| McFarland Clinic, PC | 1215 Duff Avenue | Ames | Iowa | 50010 |
| Covenant Clinic | 200 E. Ridgeway | Waterloo | Iowa | 50702 |
| IU Simon Cancer Center | 535 Barnhill Drive | Indianapolis | Indiana | 46202 |
| South Bend Clinic & Surgery Ctr | 211 N Eddy St | South Bend | Indiana | 46617-2808 |
| Elmhurst Memorial Hospital | 155 East Brush Hill Road | Elmhurst | Illinois | 60126 |
| Ingalls Memorial Hospital Cancer Research Center | One Ingalls Drive | Harvey | Illinois | 64026 |
| Rush University Medical Center | 1725 W. Harrison St. | Chicago | Illinois | 60612 |
| John H. Stroger Hospital of Cook County | 1900 W. Polk st Suite 755 | Chicago | Illinois | 60612 |
| Cancer Care & Hematology Specialists of Chicagoland-Arlington heights | 880 West Central Road | Arlington Heights | Illinois | 60005 |
| Cancer Care & Hematology Specialists of Chicagoland- Niles | 8915 West Golf Road | Niles | Illinois | 60714 |
| Cancer Care & Hematology Specialists of Chicagoland- Winfield | 25 N Windfield Road | Winfield | Illinois | 60190 |
| Straub Clinic & Hospital Inc | 888 South King Street | Honolulu | Hawaii | 96813 |
| Phoebe Putney Memorial Hospital | 417 Third Avenue | Albany | Georgia | 31701 |
| Augusta University | 1120 15th St | Augusta | Georgia | 30912 |
| Dublin Hematology and Oncology | 207 Fairview Park Drive | Dublin | Georgia | 31021 |
| Florida Cancer Specialists | 5802 State Road 54 | New Port Richey | Florida | 34652 |
| Lake County Oncology and Hematology | 4100 Waterman Way | Tavares | Florida | 32778 |
| University of Florida College of Medicine Jacksonville | 655 West 8th Street | Jacksonville | Florida | 32209 |
| Cancer Center of Central Connecticut | 100 Grand Street | New Britain | Connecticut | 06050 |
| Rocky Mountain Cancer Centers, LLP- Aurora | 1700 South Potomac Street | Aurora | Colorado | 80012 |
| Rocky Mountain Cancer Centers, LLP- Littleton | 22 West Dry Creek Circle | Littleton | Colorado | 80120 |
| Rocky Mountain Cancer Centers, LLP- Parker | 9397 Crown Crest Blvd Ste 421 | Parker | Colorado | 80138-8789 |
| Rocky Mountain Cancer Centers, LLP- Sky Ridge | 10103 Ridge Gate Parkway | Lone Tree | Colorado | 80124 |
| Rocky Mountain Cancer Centers, LLP- Lakewood | 34 Van Gordon Street | Lakewood | Colorado | 80228 |
| Rocky Mountain Cancer Centers, LLP- Longmont | 2030 W. Mountain View Avenue | Longmont | Colorado | 80501 |
| Rocky Mountain Cancer Centers, LLP- Thorton | 8820 Huron Street | Thornton | Colorado | 80260 |
| Rocky Mountain Cancer Centers, LLP- Pueblo | 3676 Parker Boulevard | Pueblo | Colorado | 81008 |
| Alta Bates Summit Comprehensive Cancer Center | 2001 Dwight Way, Herrick Campus | Berkeley | California | 94704 |
| Mercy Research | 7301 Rogers Avenue | Fort Smith | Arkansas | 72903-4100 |
| CARTI Cancer Center | 8901 Carti Way | Little Rock | Arkansas | 72205 |
| Arizona Oncology Associates, PC - HAL-Phoenix | 3330 North 2nd Street | Phoenix | Arizona | 85012 |
| Arizona Oncology Associates, PC - HAL-Scottsdale | 10460 North 92nd Street | Scottsdale | Arizona | 85258 |
| Arizona Oncology Associates , PC - HOPE | 2070 W. Rudasill Rd. | Tucson | Arizona | 85704 |
| DCH Regional Medical Center | 809 University Boulevard East | Tuscaloosa | Alabama | 35401-2029 |
