# Supplementary material for: Characterizing and prognosticating chronic lymphocytic leukemia in the elderly: prospective evaluation on 455 patients treated in the United States
Source: BMC Cancer. 2017 Mar 16;17:198. doi: 10.1186/s12885-017-3176-x (PMC5356242; doi:10.1186/s12885-017-3176-x)
Supplement: Additional file 2: Table S1. — CR and ORR of patients < 75 years versus patients ≥ 75 years enrolled in LOT1 by specific therapy. (DOCX 17 kb) [file 12885_2017_3176_MOESM2_ESM.docx]

**Table SI** CR and ORR of patients < 75 years versus patients ≥ 75 years enrolled in LOT1 by specific therapy

| Enrollment therapy | CR, % | | | ORR, % | | | | | |
| --- | --- | --- | --- | --- | --- | --- | --- | --- | --- |
|  | < 75 years  (n = 630) | ≥ 75 years  (n = 259) | *p* value | | < 75 years  (n = 630) | ≥ 75 years  (n = 259) | | *p* value | |
| BR | 48.9 | 38.5 | **0.1671** | | 63.9 | 58.5 | | **0.4580** | |
| FCR | 58.3 | 33.3 | **0.0394** | | 76.3 | 50.0 | | **0.0138** | |
| Other | 27.5 | 20.5 | **0.0916** | | 56.1 | 44.3 | | **0.0147** | |
| Breslow-Day test |  |  | **0.5129** | |  | |  | | **0.2659** |

BR bendamustine/rituximab, CR complete response, FCR fludarabine/cyclophosphamide/rituximab, LOT1 first line of therapy, ORR overall response rate
