# Supplementary material for: Characterizing and prognosticating chronic lymphocytic leukemia in the elderly: prospective evaluation on 455 patients treated in the United States
Source: BMC Cancer. 2017 Mar 16;17:198. doi: 10.1186/s12885-017-3176-x (PMC5356242; doi:10.1186/s12885-017-3176-x)
Supplement: Additional file 3: Table S2. — Cause-specific hazards analysis of prognostic indicators for OS. (DOCX 18 kb) [file 12885_2017_3176_MOESM3_ESM.docx]

**Table S2** Cause-specific hazards analysis of prognostic indicators for OS.

Patients in LOT1 stratified by death from CLL progression versus death from CLL progression or infection (*p* < 0.1)

| Baseline characteristic | Death from CLL progression | Death from CLL progression or infection |
| --- | --- | --- |
|  | Cause-specific HR (95% CI) | Cause-specific HR (95% CI) |
| del(17p) abnormality (no vs. yes) | 0.349 (0.159–0.764) | 0.343 (0.175–0.670) |
| Risk group | NS | 1.743 (0.968–3.141) |
| Site (academic vs. other) | 1.907 (0.897–4.054) | 1.847 (0.999–3.415) |
| Race (non-white vs*.*white) | NS | 1.989 (1.117–3.542) |
| Age < 75 vs. ≥ 75 years | 0.369 (0.214–0.635) | 0.350 (0.226–0.543) |
| CD38 (negative/NT vs. positive) | NS | 0.672 (0.426–1.063) |
| Geographical region: South vs. West | 0.518 (0.242–1.106) | NS |
| Enrollment therapy |  |  |
| Other therapy vs.FCR | 2.020 (0.998–4.089) | 2.020 (1.136–3.593) |
| FCR (no vs. yes) | NS | 1.781 (1.016–3.123) |
| BR or FCR (yes vs. no) | 0.508 (0.287–0.900) | 0.547 (0.347–0.863) |
| CCI score ≤ 2 vs. ≥ 3 | NS | 0.683 (0.441–1.060) |
| Insurance (other vs. private) | 1.936 (1.074–3.488) | 1.594 (1.007–2.523) |
| Anemia | 0.555 (0.317–0.970) | 0.546 (0.348–0.857) |

BR bendamustine/rituximab, CCI Charlson comorbidity index, CI confidence interval, CLL chronic lymphocytic leukemia, FCR fludarabine/cyclophosphamide/rituximab, HR hazard ratio, LOT1 first line of therapy, NS not significant, NT not specified, OS overall survival
