# Supplementary material for: Characterizing and prognosticating chronic lymphocytic leukemia in the elderly: prospective evaluation on 455 patients treated in the United States
Source: BMC Cancer. 2017 Mar 16;17:198. doi: 10.1186/s12885-017-3176-x (PMC5356242; doi:10.1186/s12885-017-3176-x)
Supplement: Additional file 5: Table S4. — Incidence of serious adverse events of grade ≥ 3 in enrolled patients by therapy and age group. (DOCX 18 kb) [file 12885_2017_3176_MOESM5_ESM.docx]

**Table S4** Incidence of serious adverse events of grade ≥ 3 in enrolled patients by therapy and age group

|  | LOT1  (N = 889) | | LOT≥2  (N = 605) | |
| --- | --- | --- | --- | --- |
| Adverse event, n (%) | < 75 years  (n = 630) | ≥ 75 years  (n = 259) | < 75 years  (n = 409) | ≥ 75 years  (n =196) |
| Patients with ≥ 1 serious adverse event of grade ≥ 3 | 219 (34.8) | 133 (51.4) | 235 (57.5) | 120 (61.2) |
| Infections |  |  |  |  |
| Pneumonia | 25 (4.0) | 25 (9.7) | 56 (13.7) | 25 (12.8) |
| Sepsis | 11 (1.7) | 7 (2.7) | 13 (3.2) | 10 (5.1) |
| Lobar pneumonia | 1 (0.2) | 2 (0.8) | 4 (1.0) | 5 (2.6) |
| Secondary neoplasms |  |  |  |  |
| Squamous cell carcinoma | 15 (2.4) | 12 (4.6) | 13 (3.2) | 7 (3.6) |
| Hematologic disorders |  |  |  |  |
| Febrile neutropenia | 22 (3.5) | 6 (2.3) | 33 (8.1) | 12 (6.1) |
| Anemia | 9 (1.4) | 9 (3.5) | 18 (4.4) | 8 (4.1) |
| Pancytopenia | 3 (0.5) | 5 (1.9) | 9 (2.2) | 3 (1.5) |
| Respiratory disorders |  |  |  |  |
| Acute respiratory failure | 5 (0.8) | 3 (1.2) | 10 (2.4) | 3 (1.5) |
| Dyspnea | 6 (1.0) | 4 (1.5) | 9 (2.2) | 2 (1.0) |
| Injection site reactions |  |  |  |  |
| Pyrexia | 21 (3.3) | 7 (2.7) | 11 (2.7) | 3 (1.5) |
| Cardiac disorders |  |  |  |  |
| Congestive cardiac failure | 3 (0.5) | 5 (1.9) | 6 (1.5) | 9 (4.6) |
| Atrial fibrillation | 3 (0.5) | 8 (3.1) | 7 (1.7) | 4 (2.0) |

NOTE. Adverse events occurring in > 2% patients in at least one patient group are listed.

LOT1 first line of therapy, LOT≥2 second line of therapy or greater
